# Comparison of Superomedial Pedicle Reduction Mammaplasty With and Without Inferior Dermal Flap Support: A One-Year Study of Ptosis and Pseudoptosis

**DOI:** 10.7759/cureus.85179

**Published:** 2025-06-01

**Authors:** Marius Kulikauskas, Kristupas A Suslavičius, Rokas Staškūnas, Kamilė Meliešiūtė, Ernest Zacharevskij

**Affiliations:** 1 Department of Plastic and Reconstructive Surgery, Lithuanian University of Health Sciences, Kaunas, LTU; 2 Faculty of Medicine, Medical Academy, Lithuanian University of Health Sciences, Kaunas, LTU

**Keywords:** breast hypertrophy, breast reduction, inferior dermal flap, inframammary fold (imf), nipple-areola complex (nac), ptosis, reduction mammaplasty, superomedial pedicle

## Abstract

Introduction: Reduction mammaplasty effectively alleviates symptoms of breast hypertrophy, but long-term ptosis and pseudoptosis remain challenges. The superomedial pedicle technique ensures reliable nipple-areola complex (NAC) vascularity and upper pole fullness, though lower pole support is limited. This study evaluates the impact of adding an inferior dermal flap on postoperative breast shape stability.

Materials and methods: This retrospective cohort study conducted at Kaunas Clinics included 54 overweight women who underwent bilateral reduction mammaplasty using a superomedial pedicle, with (Group 2, n = 26) or without (Group 1, n = 28) inferior dermal flap reinforcement. Exclusion criteria included age below 18, smoking, and systemic comorbidities. Patients were assigned to one of the two groups based on the technique: Group 1 underwent superomedial pedicle reduction without additional support, while Group 2 received the same technique with an inferior dermal flap anchored to the chest wall. Standardized anthropometric measurements (sternal notch-to-nipple (SN-N) and nipple-to-inframammary fold (N-IMF) distances) were recorded preoperatively, within 24 hours postoperatively, and at the one-year follow-up. Nipple-to-scar (n-scar) distance was measured only at the one-year follow-up. Statistical significance was set at p < 0.05.

Results: Baseline demographics and resection weights were similar between groups. Both techniques resulted in significant immediate postoperative reductions in the SN-N and N-IMF distances, with no significant intergroup differences. At one year, Group 2 showed superior lower pole support (median N-IMF: 8 cm vs. 9.5 cm, p < 0.0001) and shorter scars (median n-scar: 7.25 cm vs. 8 cm, p < 0.0001), while Group 1 retained a more stable SN-N distance (median: 21 cm vs. 22 cm, p = 0.0029).

Conclusion: The addition of an inferior dermal flap to the superomedial pedicle technique in reduction mammaplasty significantly enhances long-term lower pole stability, reduces recurrence of ptosis and pseudoptosis, and improves scar quality. This approach is especially advantageous for patients with higher BMI or reduced tissue elasticity. Despite the slightly increased operative time, the improved contour durability and aesthetic outcomes support the routine use of dermal flap reinforcement in surgical planning.

## Introduction

Reduction mammaplasty is a commonly performed surgical procedure designed to alleviate physical discomfort and enhance aesthetic appearance in patients with symptomatic breast hypertrophy [1]. Despite achieving immediate postoperative improvements, surgeons frequently encounter persistent challenges related to long-term breast shape stability, specifically ptosis and pseudoptosis. Ptosis is characterized by the descent of the nipple-areola complex (NAC) below the inframammary fold (IMF), whereas pseudoptosis refers to the sagging of the lower breast pole while the NAC remains positioned above the IMF [2-4]. Both conditions negatively impact physical comfort and breast symmetry, potentially diminishing patient satisfaction over time [5,6].

Numerous surgical techniques have been devised to address and mitigate these recurrent shape issues, with the choice of pedicle design being particularly influential in determining long-term outcomes [7,8]. The superomedial pedicle technique, in particular, has gained widespread acceptance due to its consistent vascularization of the NAC, effective nipple elevation, and superior projection, all of which contribute significantly to reduced rates of complications such as tissue necrosis and inadequate upper pole fullness [9-11]. The integration of an inferior dermal flap has been proposed as a supplementary structural support to enhance lower pole stability and potentially improve the durability of postoperative results [12].

Despite advancements in surgical techniques, achieving consistent long-term correction of ptosis and pseudoptosis remains challenging [13]. Studies have shown that while the initial results after surgery are often very satisfying, factors such as tissue quality, effects of gravity, and aging can lead to recurrent sagging over time [1,14]. Consequently, contemporary research has increasingly focused on developing hybrid techniques, combining traditional pedicle methods with additional internal structural supports, aimed at maintaining long-term breast shape integrity [12,15].

This study aims to compare the effectiveness of superomedial pedicle techniques with and without dermal flap support in reducing ptosis and pseudoptosis over a one-year follow-up period. By analyzing the degree of recurrence in ptosis and pseudoptosis, we seek to provide evidence-based guidance for surgeons to optimize surgical planning in breast reduction procedures.

## Materials and methods

Study design

**Figure 1:**
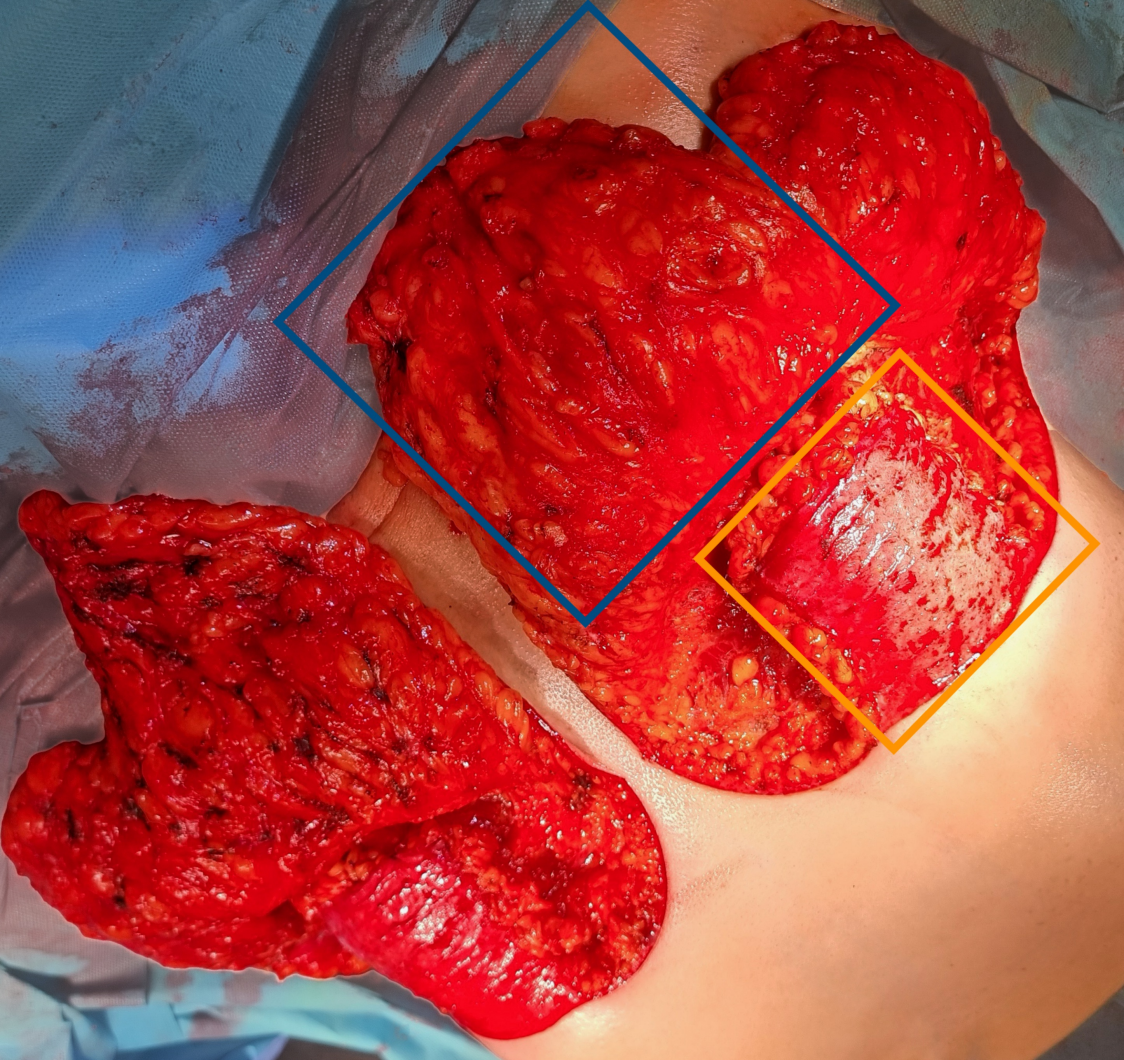
Intraoperative view of both breasts using superomedial pedicle technique with an inferior dermal flap The inferior dermal flap (yellow box) and superomedial pedicle (blue box) are highlighted on the left breast.

This retrospective cohort study included female patients who underwent bilateral reduction mammaplasty for symptomatic breast hypertrophy. Patients under 18 years of age, with a history of smoking, or with comorbidities such as systemic connective tissue diseases, diabetes mellitus, or ischemic heart disease were excluded. Additionally, during the one-year follow-up, patients who experienced a postoperative body weight change of more than 3 kg, excluding the intraoperative weight of excised breast tissue, were excluded to minimize the influence of systemic weight fluctuations on breast shape outcomes and maintain group homogeneity. Individuals with known hypersensitivity to lidocaine or epinephrine were also excluded. All procedures were performed at the Department of Plastic and Reconstructive Surgery at Kaunas Clinics, following a standardized day-care protocol. Surgeries were conducted under general anesthesia by a single experienced plastic surgeon. Eligible patients were randomized into two groups using block randomization with a block size of four to ensure balanced group sizes throughout the enrollment period. Allocation was concealed using sequentially numbered, sealed, opaque envelopes prepared by an independent staff member not involved in the surgeries or assessments. Group 1 (n = 26 patients, 56 breasts) underwent reduction mammaplasty using a superomedial pedicle technique without additional support. Group 2 (n = 26 patients, 52 breasts) underwent the same superomedial pedicle technique supplemented with an inferior dermal flap fixed to the chest wall for added structural support (Figure 1). Demographic data, including age and body mass index (BMI), were recorded preoperatively. The total weight of the resected breast tissue was measured intraoperatively for each breast.

Surgical technique

A standard Wise-pattern incision was utilized for all patients. Both groups underwent glandular tissue resection primarily from the inferior and lateral quadrants while preserving optimal vascularity and sensation of the NAC through a superomedial parenchymal pedicle. In Group 2, following glandular resection, an inferiorly based dermal flap was fashioned from the de-epithelialized lower pole skin, measuring approximately 8 × 8 cm, and centered symmetrically along the projected nipple midline. The flap was designed to preserve subdermal vascular integrity and was anchored at five strategic points (bilateral superior corners, lateral margins) to the pectoralis major fascia at the level of the newly established inframammary fold. Fixation was performed using interrupted 2-0 polyglactin (Vicryl) sutures to ensure uniform distribution of mechanical support across the lower breast pole. Care was taken to avoid excessive tension. Once secured, the remaining breast parenchyma was reshaped, and the skin was closed in layers as per the standard Wise-pattern technique. Surgical drains were routinely placed and removed when the output decreased to clinically acceptable levels.

Anthropometric measurements

Standardized anthropometric measurements were performed consistently by the same surgeon using a flexible measuring tape, with patients standing upright. Measurements were recorded preoperatively, within 24 hours postoperatively, and at the one-year follow-up. Key measurements included distances from the sternal notch to the nipple (SN-N), from the nipple to the inframammary fold (N-IMF), and from the nipple to the scar (n-scar; measured from the nipple center to the midpoint of the vertical limb of the inverted-T scar, recorded only at the one-year follow-up). The primary outcome measures evaluated were changes in SN-N and N-IMF distances from immediately postoperative to one-year follow-up, comparing results between Groups 1 and 2.

Data analysis

Collected data were entered into a Microsoft Excel spreadsheet (Microsoft Corp., Redmond, WA, US) and analyzed using GraphPad Prism software version 10.3.1 (Dotmatics, Boston, MA, USA). Data normality was assessed using Shapiro-Wilk and Kolmogorov-Smirnov tests. Descriptive statistics were reported using means (±) and median values (min-max) for continuous variables. For comparisons between the two groups, the independent samples t-test was used for normally distributed data, while the Mann-Whitney U test was applied to non-normally distributed data. All statistical analyses were performed using two-tailed tests, with a significance threshold set at p < 0.05 for hypothesis testing. The study's findings were presented using graphs and tables.

Ethical approval

This study was approved by the Bioethics Committee of the Lithuanian University of Health Sciences (approval number BEC2-954). All participants were informed about the study’s objectives and assured of the confidentiality of their data and responses.

## Results

A total of 54 female patients (108 breasts) met the inclusion criteria and were divided into two groups: 28 patients (56 breasts) in Group 1 (superomedial pedicle without dermal flap) and 26 patients (52 breasts) in Group 2 (superomedial pedicle with inferior dermal flap).

There were no statistically significant differences in demographic characteristics between the two groups. All participants were classified as overweight based on their BMI. Specifically, Group 1 had a median age of 47 years (range: 24-73) and a median BMI of 28.84 (range: 25.83-35.32), while Group 2 had a median age of 48 years (range: 40-58) and a median BMI of 30.48 (range: 24.57-35.32) (age: p = 0.138; BMI: p = 0.1384). The median resected breast tissue mass was also similar between the groups: 936 g (range: 438-2000 g) in Group 1 and 803 g (range: 386-1740 g) in Group 2 (p = 0.5323) (Table 1).

**Table 1:** Demographic characteristics

Group	Group 1	Group 2	P-value
Patients, n (breasts)	28 (56)	26 (52)	
Age, median (range)	47 (24-73)	48 (40-58)	0.138
BMI, median (range) (kg/m²)	28.63 (25.83-35.32)	30.48 (25.14-35.32)	0.1384
Resected breast mass, median (range) (g)	936 (438–2000)	803 (386–1740)	0.5323

Preoperatively, Group 1 exhibited a median SN-N distance of 31 cm (range: 27-42 cm) and a median N-IMF distance of 14 cm (range: 10-20 cm). Group 2 showed comparable measurements, with a median SN-N distance of 32.75 cm (range: 28-40 cm) and a median N-IMF distance of 15 cm (range: 10-20 cm). No statistically significant preoperative differences were observed between the two groups (SN-N: p = 0.5609; N-IMF: p = 0.8941) (Table 2).

**Table 2:** Comparison of anthropometric measurements between groups SN-N: sternal notch-to-nipple distance, N-IMF: nipple-to-inframammary fold distance; n-scar: nipple-to-scar distance. *Statistically significant result at p < 0.05.

Group	Group 1	Group 2	
Variable	Measurement, median (range)	Measurement, median (range)	p-value
SN-N (cm)			
Before surgery	31 (27-42)	32.75 (28-40)	0.5609
Within 24 hours after surgery	21 (19.5-24)	22 (20-22)	0.1928
One year after surgery	21 (20.5-24)	22 (20-24)	0.0029*
N-IFM (cm)			
Before surgery	14 (10-20)	15 (10-20)	0.8941
Within 24 hours after surgery	6.5 (5.5-8)	6.5 (5.5-7)	0.1253
One year after surgery	9.5 (8.5-12.5)	8 (7-11)	<0.0001*
n-scar (cm)	8 (7-10)	7.25 (6-9)	<0.0001*

Immediately postoperatively (within 24 hours), both techniques resulted in significant reductions in SN-N and N-IMF distances. Group 1 showed a median SN-N distance reduction to 21 cm (range: 19.5-24 cm) and median N-IMF distance reduction to 6.5 cm (range: 5.5-8 cm). Similarly, Group 2 showed a median SN-N distance reduction to 22 cm (range: 20-22 cm) and a median N-IMF distance reduction to 6.5 cm (range: 5.5-7 cm). At this early postoperative point, the differences between groups were not statistically significant (SN-N: p = 0.1928; N-IMF: p = 0.1253) (Table 2).

**Figure 2:**
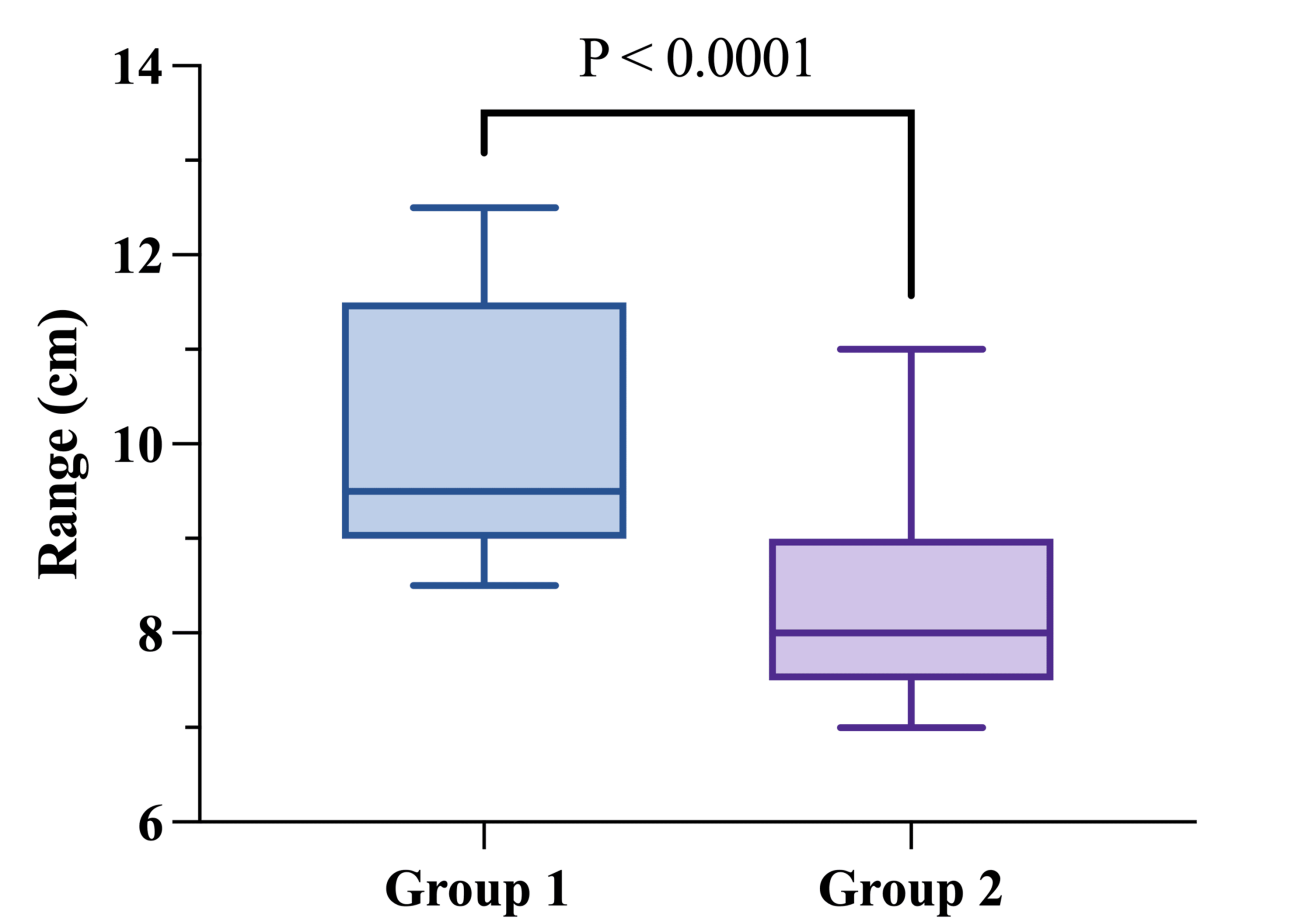
N-IMF one year after operation N-IMF: nipple-to-inframammary fold distance. Statistical comparison was performed using Mann-Whitney U. Statistical significance was defined as p < 0.05.

At the one-year postoperative follow-up, significant differences emerged between the groups. The SN-N distance in Group 1 remained stable with a median of 21 cm (range: 20.5-24 cm), while Group 2 showed a slightly longer median SN-N distance of 22 cm (range: 20-24 cm), a statistically significant difference (p = 0.0029) (Table 2). However, Group 2 exhibited significantly better stability of the lower pole, with a median N-IMF distance of 8 cm (range: 7-11 cm) compared to a median N-IMF of 9.5 cm (range: 8.5-12.5 cm) in Group 1 (p < 0.0001) (Figure 2).

**Figure 3:**
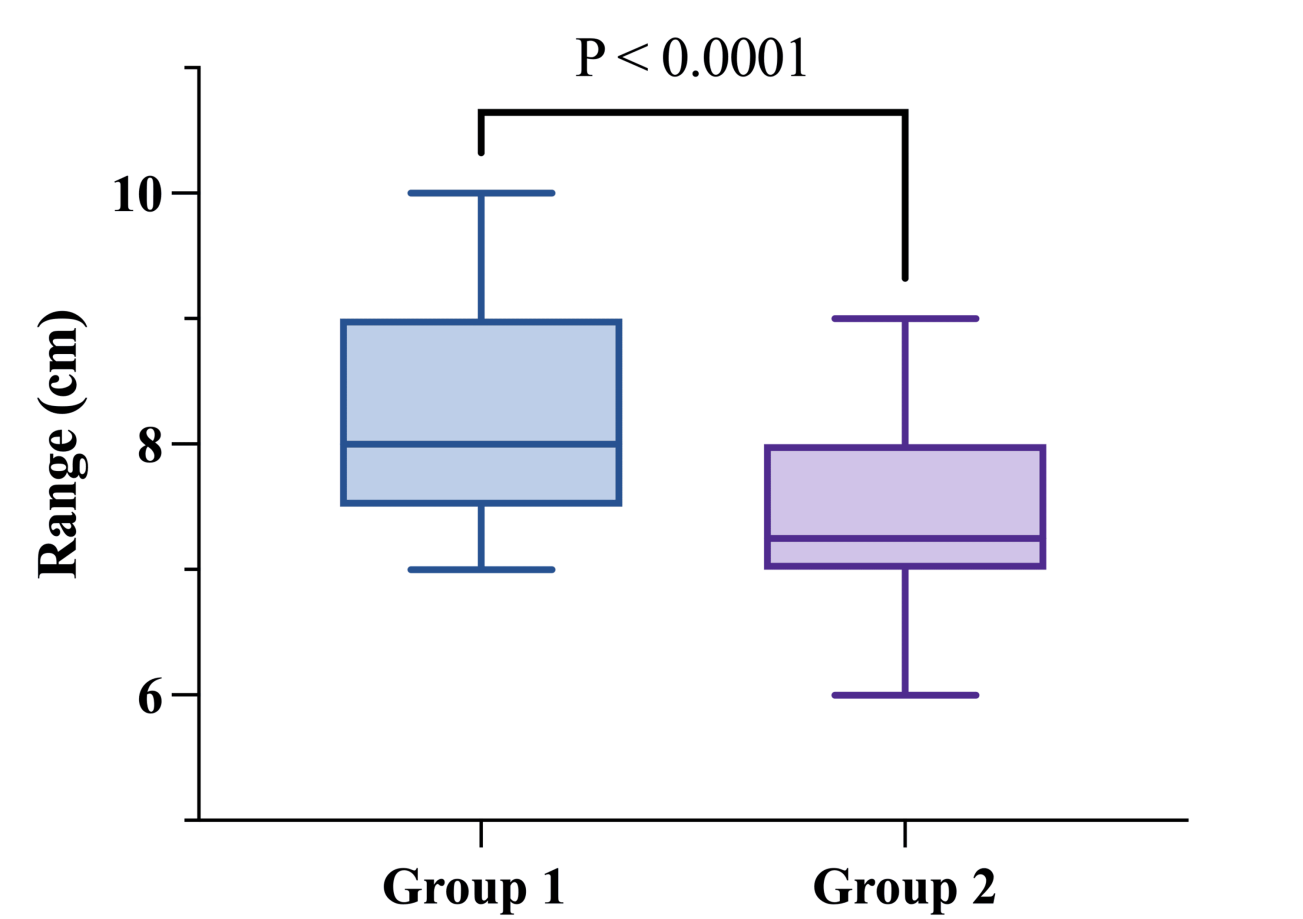
n-scar one year after operation n-scar: nipple-to-scar distance. Statistical comparison was performed using Mann-Whitney U. Statistical significance was defined as p < 0.05.

Scar position, measured as the n-scar distance at the one-year follow-up, was significantly shorter in Group 2 (median 7.25 cm, range: 6-9 cm) compared to Group 1 (median 8 cm, range: 7-10 cm) (p < 0.0001) (Figure 3). This finding highlights the positive impact of dermal flap reinforcement on improving scar outcomes after reduction mammaplasty (Table 2).

## Discussion

The findings of this study demonstrate that incorporating inferior dermal flap support into the superomedial pedicle technique significantly enhances lower pole stability and effectively reduces the recurrence of ptosis and pseudoptosis. The significantly smaller increase in N-IMF distance observed in patients treated with the combined superomedial pedicle and inferior dermal flap support compared to those treated with the superomedial pedicle technique alone highlights the efficacy of the dermal flap in maintaining breast contour over time. These findings align closely with the systematic review by Wagner et al., who concluded that superior and superomedial pedicle techniques, especially when supplemented with supportive structures such as dermal flaps or muscular slings, offer superior long-term breast shape stability compared to inferior pedicle techniques alone [16].

Our findings regarding the efficacy of the superomedial pedicle technique with inferior dermal flap support align with the work of Gargano et al. on dermoglandular flaps in breast reduction, specifically his "yin-yang technique" [17]. While our study focused on comparing superomedial pedicle techniques with and without dermal flap support, their approach similarly emphasizes the importance of structural reinforcement for maintaining breast shape and preventing ptosis. Gargano et al. reported excellent outcomes with their technique, with 95% of patients pleased with their results in terms of breast size, shape, and scar appearance, which corresponds with our findings of improved lower pole stability, support, and reduced recurrence of ptosis [17].

Our study also demonstrated a significant difference in scar position between the two groups, with notably shorter n-scar distances observed in patients who received dermal flap support. This finding underscores the beneficial role of the dermal flap in improving scar aesthetics by redistributing mechanical tension away from the primary incision site. By decreasing tissue tension during closure, the dermal flap contributes to the formation of shorter, less conspicuous, and more cosmetically acceptable scars [17,18]. Kemaloğlu et al. similarly reported that structural reinforcement techniques indirectly improve scar quality by limiting tissue stretch and stress during wound healing [19]. Although aesthetic outcomes were not the primary focus of this study, enhanced scar quality remains an important factor that could significantly improve patient satisfaction, particularly in individuals who place high importance on cosmetic outcomes [20].

Our results suggest that the inferior dermal flap technique could be particularly advantageous for patients with increased BMI or diminished tissue elasticity, groups typically at higher risk for recurrence of breast ptosis. Stabilizing the lower pole effectively addresses one of the most common and challenging postoperative complications following reduction mammaplasty [21,22]. This technique thus represents an effective approach for surgeons aiming to achieve durable aesthetic and functional outcomes without compromising the safety or vascularity of the NAC [23]. The use of the inferior dermal flap in our technique shares similarities with approaches used in skin-reducing mastectomies, where the dermal flap reinforces the inferior lateral seat of implants and provides additional structural support [24]. The addition of the dermal flap procedure offers long-term benefits, including improved breast contour stability and enhanced aesthetic longevity, which justify this additional surgical step, particularly in patients predisposed to postoperative recurrence of ptosis [25]. Additionally, our findings support the outcomes of the dermal sling suspension technique introduced by Loonen and Tahir, who described reduced ptosis through structural reinforcement of the superior pedicle [12].

Extensive literature supports our observations, demonstrating the superiority of the superomedial pedicle technique over other techniques in terms of clinical and aesthetic outcomes. Comparative studies have shown that patients undergoing superomedial pedicle reduction are 4.3 times less likely to experience postoperative complications than those undergoing inferior pedicle reduction, with significantly better scarring scores and shorter procedure times. This advantage becomes particularly important in cases of gigantomastia, where the risk of complications traditionally increases with larger resection volumes [26,27].

## Conclusions

The findings of this study support the integration of an inferior dermal flap with the superomedial pedicle technique in reduction mammaplasty. This combination significantly improves long-term lower pole stability, effectively reduces the recurrence of ptosis and pseudoptosis, and enhances scar quality by decreasing mechanical tension at the incision site. The use of the inferior dermal flap is particularly beneficial for patients at increased risk of recurrent sagging due to higher BMI or reduced tissue elasticity. Despite slightly prolonged operative times, the substantial benefits observed in breast contour durability and cosmetic outcomes provide strong justification for routinely incorporating inferior dermal flap reinforcement into surgical planning for reduction mammaplasty.
